# *In silico* Investigations of the Mode of Action of Novel Colchicine Derivatives Targeting β-Tubulin Isotypes: A Search for a Selective and Specific β-III Tubulin Ligand

**DOI:** 10.3389/fchem.2020.00108

**Published:** 2020-02-21

**Authors:** Lorenzo Pallante, Antonio Rocca, Greta Klejborowska, Adam Huczynski, Gianvito Grasso, Jack A. Tuszynski, Marco A. Deriu

**Affiliations:** ^1^PolitoBIOMed Lab, Department of Mechanical and Aerospace Engineering, Politecnico di Torino, Turin, Italy; ^2^Department of Chemistry, Adam Mickiewicz University, Poznań, Poland; ^3^Dalle Molle Institute for Artificial Intelligence (IDSIA), University of Applied Sciences of Southern Switzerland (SUPSI), University of Italian Switzerland (USI), Manno, Switzerland; ^4^Department of Oncology, University of Alberta, Edmonton, AB, Canada

**Keywords:** molecular modeling, drug discovery, microtubule, cancer, drug resistance, tubulin, colchicine, colchicine derivatives

## Abstract

The cardinal role of microtubules in cell mitosis makes them interesting drug targets for many pharmacological treatments, including those against cancer. Moreover, different expression patterns between cell types for several tubulin isotypes represent a great opportunity to improve the selectivity and specificity of the employed drugs and to design novel compounds with higher activity only on cells of interest. In this context, tubulin isotype βIII represents an excellent target for anti-tumoral therapies since it is overexpressed in most cancer cells and correlated with drug resistance. Colchicine is a well-known antimitotic agent, which is able to bind the tubulin dimer and to halt the mitotic process. However, it shows high toxicity also on normal cells and it is not specific for isotype βIII. In this context, the search for colchicine derivatives is a matter of great importance in cancer research. In this study, homology modeling techniques, molecular docking, and molecular dynamics simulations have been employed to characterize the interaction between 55 new promising colchicine derivatives and tubulin isotype βIII. These compounds were screened and ranked based on their binding affinity and conformational stability in the colchicine binding site of tubulin βIII. Results from this study point the attention on an amide of 4-chlorine thiocolchicine. This colchicine-derivative is characterized by a unique mode of interaction with tubulin, compared to all other compounds considered, which is primarily characterized by the involvement of the α-T5 loop, a key player in the colchicine binding site. Information provided by the present study may be particularly important in the rational design of colchicine-derivatives targeting drug resistant cancer phenotypes.

## Introduction

The pivotal role of microtubules (MTs) in the mitotic process make them important targets for anticancer therapies since cancerous cells proliferate by unregulated cell division (Gajewski et al., 2013). By either stabilizing MTs or enhancing their depolymerization, it is possible to halt the mitotic process and eventually lead cells to apoptosis (Nettles et al., 2002). Among antimitotic agents, colchicine is able to block cell division (Bhattacharyya et al., 2008) by destabilizing MT assembly kinetics and dynamics. In particular, when colchicine binds in its specific binding site (located at the interface between tubulin α and β monomers) the structural conformation of the tubulin dimer is affected in such a way that tubulin integration into the MT lattice is hampered.

However, one of the main drawbacks of colchicine is its general toxicity (Wallace, 1974; Finkelstein et al., 2010). Several studies in the past have proposed less toxic colchicine derivatives as an alternative to colchicine (Lu et al., 2012; Wang et al., 2016; Johnson et al., 2017; Majcher et al., 2018a,b; Klejborowska et al., 2019). Moreover, these novel colchicine derivatives may be designed to show high specificity only for tubulin isotypes, which are over-expressed in cancer, in order to maximize their effect only on tumor cells and reduce side effects of the drug due to its toxicity on normal cells (Lu and Luduena, 1994; Luduena et al., 1995).

Differing in point or restricted sequence variations, several tubulin isotypes (Leandro-García et al., 2010) are differently expressed by cells under both physiological and pathological conditions. For example, tubulin isotype αβIII is considered as an excellent target for anti-tumoral therapies because it is over-expressed in tumoral cells and it is less widespread than other isotypes, such as αβI, αβII and αβIV, in normal cells (Ferlini et al., 2007; Tseng et al., 2010). Moreover, an over-expression of tubulin isotype αβIII by cancer cells is considered as one among several known drug resistance mechanisms (Derry et al., 1997; Ludueña, 1998; Katsetos et al., 2003; Kamath et al., 2005; Seve, 2005; Ferlini et al., 2007; Sève and Dumontet, 2008; Tseng et al., 2010). Thereby, it is of primary importance to identify specific compounds, which selectively target isotype αβIII.

In this context, computational molecular modeling techniques, such as molecular dynamics (MD) and molecular docking, represent powerful tools to shed light on the molecular mechanisms concerning protein functions and their interaction between different ligands and a specific receptor (Lepre et al., 2017; Omar et al., 2018; Brogi, 2019; Sirous et al., 2019). These computational methods can be applied to investigate the action of different ligands on tubulin dimers (Mitra and Sept, 2008; Natarajan and Senapati, 2012; Gajewski et al., 2013; Kumbhar et al., 2016). Computational drug discovery may help to accelerate and economize the drug discovery process as a complementary tool for experimental research of novel inhibitors.

In this work, ensemble molecular docking, molecular dynamics simulations, and binding energy estimation methods have been employed to characterize the binding of 55 novel colchicine derivatives to the βIII tubulin isotype. We have identified an interesting 4-chlorine thiocolchicine derivative characterized by similar affinity but a different mode of binding to tubulin with respect to its parent compound, colchicine. The main findings of our study indicate this ligand as a promising candidate to overcome colchicine drawbacks and provide information for further developments in designing more selected and specific colchicine derivatives with an intended use as cancer chemotherapy agents.

## Materials and Methods

### Atomic Models of Investigated Compounds

Several series of novel colchicine derivatives (Majcher et al., 2018a,b; Klejborowska et al., 2019) were considered in this work. All 55 compounds have shown *in vitro* anti-proliferative effects on normal and cancer cells. In particular, they were tested on human lung adenocarcinoma, human breast adenocarcinoma, human colon adenocarcinoma cell lines and a doxorubicin-resistant subline (Majcher et al., 2018a,b; Klejborowska et al., 2019).

**Figure 1:**
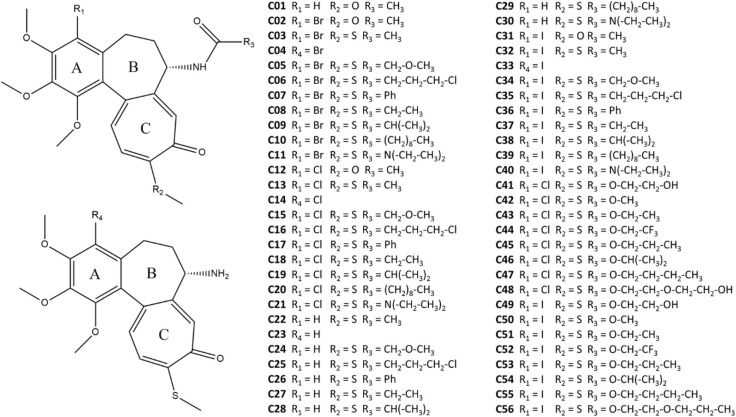
Colchicine and its derivatives considered in this work.

These compounds can be divided into five classes: 4-Br-Amides (10 compounds), 4-Cl-Amides (10 compounds), DT-and-4I-Amides (19 compounds), 4-Cl-Carbamates (8 compounds) and 4-I-Carbamates (8 compounds). The chemical structures of colchicine (C01) and its derivatives (C02-C56) are summarized in Figure 1.

The 2D structures of the colchicine derivatives have been drawn using ChemDraw 12.0, whereas their 3D structure was designed by AVOGADRO (Hanwell et al., 2012).

### Human αβIII Tubulin Modeling and Conformational Dynamics

The atomic structures of human βIII tubulin isotype were obtained by homology modeling, starting from the Protein Data Bank (PDB) entry 4O2B model (Prota et al., 2014) as a template. This structure was chosen due to its high resolution (2.3 Å) and a low number of missing residues (Aryapour et al., 2017). First, from the starting template the information concerning tubulin αβ, GTP, GDP, Mg^2+^ ion and colchicine was extracted. Missing residues in β tubulin (from 276 to 281) were added by MODELER 9.20 (Šali and Blundell, 1993) where the best model was selected on the basis of the obtained DOPE (Discrete optimized protein energy) score. Then, the Fasta sequences Q71U36 and Q13509 were selected from the Uniprot website, respectively, for the α and β subunits. The above-mentioned amino acid sequences pertain to the isotype αβIII (Gajewski et al., 2013; Kumbhar et al., 2016). Homology modeling was then employed by MODELER 9.20 to generate a 3D structure of the αβIII sequence using the 4O2B model. The quality and the reliability of the generated model were evaluated using PROCHECK (Laskowski et al., 1993), VERIFY3D (Colovos and Yeates, 1993) and ERRAT (Bowie et al., 1991), as reported in previous literature in this area (Huzil et al., 2006; Deriu et al., 2007; Mane et al., 2008; Kumbhar et al., 2016).

Two systems were subsequently considered: (I) tubulin, GTP, GDP, and Mg^2+^ ion and (II) tubulin, GTP, GDP, Mg^2+^ ion and colchicine bound to tubulin. Information on colchicine binding was taken from the 4O2B model. The AMBER ff99SB-ILDN forcefield (Lindorff-Larsen et al., 2010) was used to describe protein, water and ion topology. GTP, GDP, and ligands were described by the General Amber Force Field (GAFF) (Wang et al., 2004) and AM1-BCC charge method (Jakalian et al., 2002), as applied in many previous studies (Gajewski et al., 2013; Kumbhar et al., 2016; Klejborowska et al., 2019; Sahakyan et al., 2019).

Each of the above mentioned protein systems (I and II) was then inserted in a dodecahedron box filled with TIP3P explicit water molecules (Mark and Nilsson, 2001), sodium and chlorine ions (150 mM). Particle-mesh Ewald (PME) method (Darden et al., 1993) was used to treat electrostatics (cut-off = 1.0 nm) whereas Van der Waals (VdW) interactions were treated by a plain cut-off at 1 nm (Natarajan and Senapati, 2012; Natarajan et al., 2013; Bueno et al., 2018). Each system was then energy minimized by the steepest descent algorithm for 1,000 steps with a maximum force of 100 kJmol^−1^nm^−1^. All systems were simulated in an NVT and NPT ensemble with position restraints applied on protein and ligand atoms. In detail, a 100 ps position restrained MD simulation in the NVT ensemble (Bussi et al., 2007), was followed by a 300 ps position restrained MD in the NPT (*T* = 300 K, *P* = 1 bar) ensemble (Berendsen et al., 1984; Bussi et al., 2007).

Temperature and pressure were controlled by weak coupling algorithms (Berendsen et al., 1984; Bussi et al., 2007).

Finally, production MD simulations (without restraints) were carried out for 100 ns in presence and in absence of colchicine, respectively. Ten configurations of each system were extracted as representative of structural equilibrium. The above-mentioned system configurations were then used for ensemble docking procedure.

### Ensemble Docking and Binding Energy Refinement

Ensemble docking was performed using AUTODOCK VINA 1.1.2 (Trott and Olson, 2010). The center of the search space was defined by taking, from the 4O2B model, the relative position of the colchicine in its binding site. The docking was performed using a grid space 2 × 2 × 2 nm around the center of the search space and an exhaustiveness equal to 64 was set. Each compound was docked to the ten different isotype configurations extracted from the production MD, as explained above.

Then, for each VINA pose, the binding energy refinement was performed by running short 1 ns MD simulations on the ligand-protein complex starting from the VINA best pose for each considered ligand. Each complex was followed by solvation, neutralization, energy minimization, position restrained MD, and short production MD. Simulation set up was the same as described in the previous section. On the last 100 ps of MD production the ligand-protein binding was evaluated using two criteria. Firstly, the binding energy was quantified by the Molecular Mechanics Generalized Born Surface Area (MMGBSA) method (Genheden and Ryde, 2015). The parameters were set according to the previous literature (Nguyen et al., 2013, 2015; Su et al., 2015). Secondly, the ligand conformational displacement in the binding site was quantified by calculating the Root Mean Square Deviation (RMSD) of ligand carbon rings (a common feature of all considered compounds with colchicine). In particular, for each ligand, the MD protein trajectory was fitted on a reference structure (the starting configuration of the colchicine-protein complex). In this way, the RMSD quantifies the relative deviation of each ligand with respect to the colchicine starting position throughout the overall MD trajectory. Based on the above-mentioned criteria, the best colchicine derivative and colchicine, both bound to the βIII isotype, were simulated for 100 ns in order to highlight binding conformational differences at equilibrium.

All MD simulations were carried out using GROMACS 2018.3 (Abraham et al., 2015). The Visual Molecular Dynamics (VMD) package was employed for the visual inspection of the simulated systems (Humphrey et al., 1996). Dedicated GROMACS tools were used for a quantitative analysis in terms of Root-Mean-Square Deviation (RMSD), Root-Mean-Square Fluctuation (RMSF), and clustering, while analysis of the secondary structure was performed by applying the STRuctural IDEntification (STRIDE) algorithm (Heinig and Frishman, 2004).

## Results

### Human αβIII Tubulin Model Development and Conformational Dynamics

The Ramachandran plot (see also Figure S1) obtained by PROCHECK highlighted the 95.6% of residues in most favored regions, 4.2% in additional allowed regions, and 0.1% in generously allowed regions. No residues were found in disallowed regions. Since a good quality model is expected to have at least 90% of the residues in the most favored regions (Santoshi and Naik, 2014), the built model was considered reliable. Moreover, the Overall Quality Factor obtained by the ERRAT tool for the isotype αβIII was 80.29 for the α and 84.73 for the β tubulin monomer model. It is worth mentioning that the generally accepted range is higher than 50 for a high quality model (Messaoudi et al., 2013). Finally, the VERIFY3D confirmed that 98.15% of residues showed an averaged 3D-1D score higher than 0.2 (Messaoudi et al., 2013).

First, the backbone RMSD was calculated for isotype βIII both in presence and in absence of colchicine during the overall MD simulation (100 ns): all the simulated structures reached structural equilibrium, with values under 0.3 nm (see also Figure S2). Moreover, the cluster analysis on the last 50 ns of the simulations highlighted only one cluster using an RMSD cut-off of 0.15 nm, indicating a strong stability of the simulated systems. Moreover, the cluster analysis indicated that the colchicine presence did not modify significantly the conformation of the interaction site.

### Ensemble Docking and Binding Energy Calculation

**Figure 2:**
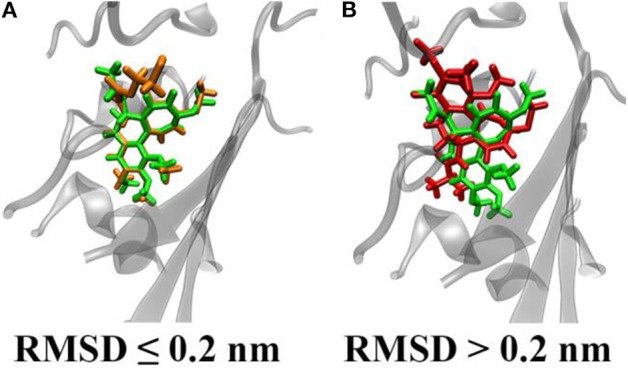
Representative snapshots of ligand conformational displacements in the colchicine binding site. Colchicine is represented in green, whereas two different derivatives with low **(A)** and high **(B)** RMSD with respect to the colchicine starting pose are depicted orange and red, respectively.

The 55 colchicine derivatives were docked to ten different configurations of βIII tubulin, extracted from the last 50 ns of the MD simulation described above. Only the best ligand pose in terms of binding affinity was considered (see also Figure S3). In order to take into account also the dynamic nature of the binding process, we have performed a MD simulation of 1 ns for each ligand-receptor complex. Throughout the quick MD run, the binding energy was quantified by means of the MM-GBSA method (Huzil et al., 2010; Gajewski et al., 2013; Kumbhar et al., 2016). Moreover, the ligand displacement in the binding site was quantified by the RMSD calculated as described in Materials and Methods. It is worth mentioning that low RMSD values indicate a compound which is stable in a spot close to the starting colchicine position, whereas high RMSD values identify a compound moving further apart (Figure 2). Most compounds showed RMSD lower than 0.2 nm, suggesting that the derivatives investigated here behaves similarly to colchicine (highly stable in its binding site during the short MD run). The only exception found is represented by compound C19 which displays high variation from the colchicine starting position (RMSD = 0.47 nm).

**Figure 3:**
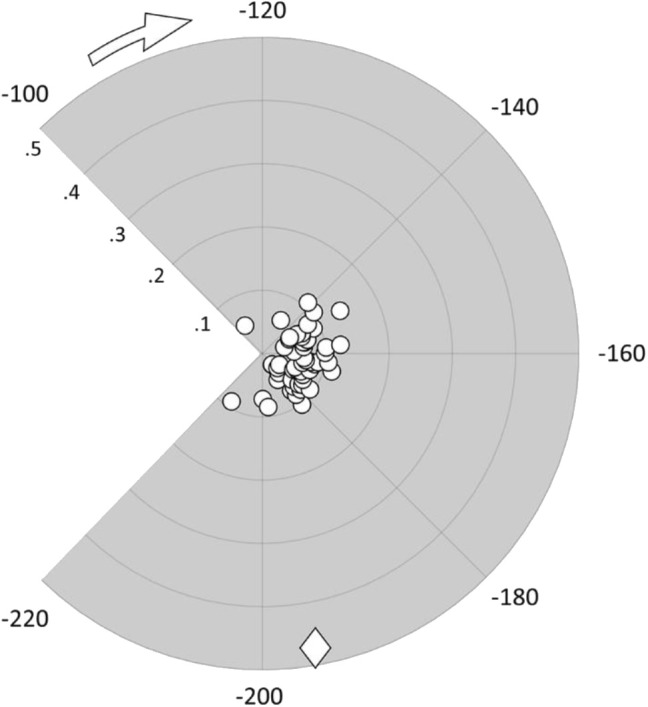
Polar scatter plot representing ligands' RMSD from the colchicine starting pose (radial coordinate) in nm and their binding energy (angular coordinate) in kJ/mol; the diamond marker represents the C19 compound.

Our binding energy analysis highlights four specific compounds (C19, C20, C29, and C48) as possible hits. In fact, they exhibit similar values of their binding energy for βIII tubulin compared to colchicine. All binding energy values are reported in Supporting Information text (see also Figure S4). In order to better describe differences between investigated compounds, we have merged RMSD and binding energy information in a single plot (Figure 3).

Interestingly, compound C19 features a peculiar behavior, i.e., it exhibits a large deviation from the colchicine starting position in the binding site (high RMSD value), with a significant difference to all the other compounds. It is also characterized by binding energy values comparable to colchicine and higher than most other derivatives. This result points the attention on the compound C19 as a promising candidate able to bind strongly to βIII human tubulin with a different mode of action with respect to colchicine.

### Conformational Dynamics of Colchicine and C19 Bound to βIII Human Tubulin

Conformational dynamics of the C19-tubulin complex has been investigated by a 100 ns long MD simulation. For comparison, a 100 ns-long MD was also carried out on the colchicine-tubulin complex. Systems were replicated to confirm the consistency of the data (Figures S5, S7).

**Figure 4:**
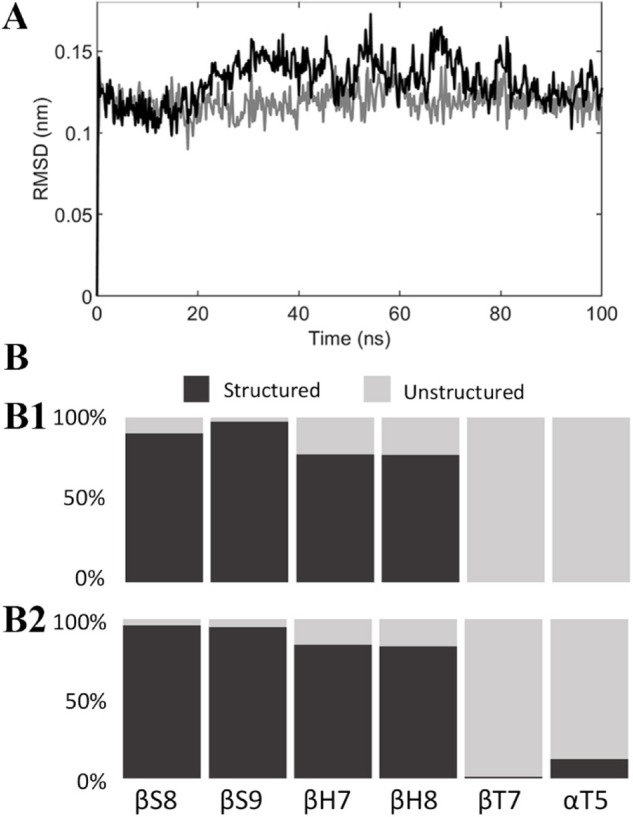
**(A)** RMSD of the colchicine binding site from its starting position when colchicine (black) or compound C19 (gray) are bound to the tubulin dimer. **(B)** Secondary structure probability of residues in the colchicine binding site when colchicine **(B1)** or compound C19 **(B2)** are bound to the tubulin dimer.

Structural modifications of the colchicine binding site were first analyzed by computing the RMSD of the tubulin binding cleft, i.e., residues within 1 nm from the ligand, from its starting position and the secondary structure probability during the last 50 ns of the simulation (Figure 4). A second replica was performed to ensure the repeatability of the results (see also Figure S5). The binding site was characterized by a structural stability throughout the overall MD, exhibiting low RMSD values (lower than 0.18 nm) and highly conserved secondary structures. The only noteworthy difference is represented by the α-T5 loop, which exhibits tendency to rearrange in a more structured shape only in the presence of C19.

**Figure 5:**
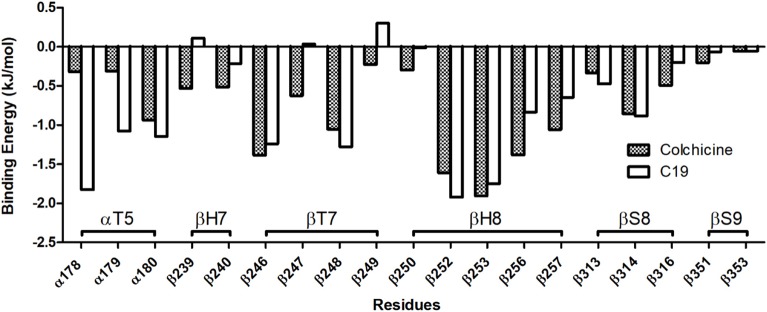
Binding Energy decomposition over the residues of the colchicine binding site (only residues with the highest energies are reported). Compound C19 has a significantly higher affinity than colchicine for the αT5 loop.

The binding energy was estimated by the MM-GBSA approach in order to compare the binding affinities of the analyzed compounds at the structural equilibrium: again, compound C19 and colchicine showed similar binding energy for isotype αβIII, respectively, −229.98 ± 22.26 kJ/mol and −223.70 ± 22.31 kJ/mol (see also Figure S6). Nevertheless, the energy decomposition over the tubulin binding cleft residues reveals that the compound C19 shows a higher binding energy compared to the colchicine for residues 178–180 of the α tubulin, which belong to the αT5 loop (Figure 5).

**Figure 6:**
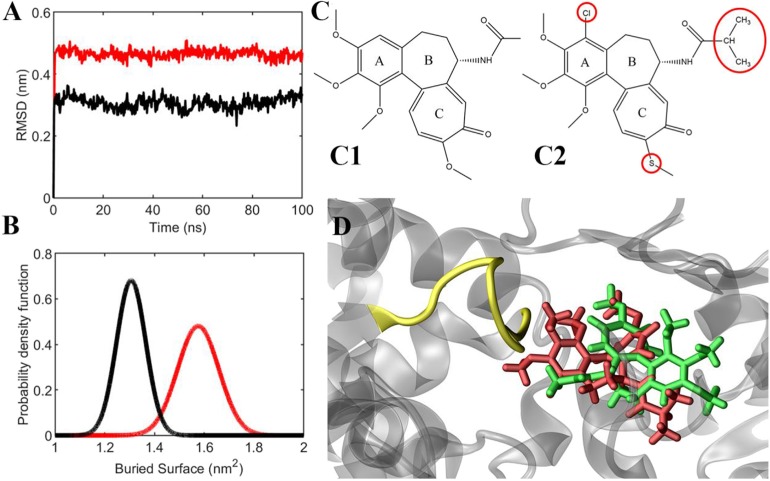
**(A)** Ligands' RMSD from their starting position (colchicine in black, C19 in red). **(B)** Probability density function of the buried surface between the ligands and the αT5 loop (colchicine in black, C19 in red), averaged between two replicas during the last 20 ns of simulation. **(C)** Chemical structures of colchicine **(C1)** and compound C19 **(C2)**. **(D)** representative snapshot of the simulation, which shows that compound C19 (red) is closer to αT5 loop (yellow) than colchicine (green).

In light of these results, the ligands' behavior in the binding site and their interaction with the αT5 loop were investigated in more depth (Figure 6). First, ligand RMSD (used to quantify the ligand movement in the binding site throughout the simulation) showed that compound C19 has a more marked tendency than colchicine to move apart, reaching a more favorable pose for the interaction with the αT5 loop (Figure 6A and see also Figure S7). Second, the interaction surface between each ligand and the αT5 loop, which quantifies the available area for their binding, is higher for C19 than colchicine (Figure 6B). Figures 6C1,C2 and 6D represent ligand structures and their relative position in the tubulin binding cleft (see also Movies S1, S2).

In conclusion, compound C19 was shown to be stable in the tubulin binding site with a relative position differing from the colchicine site. Specifically, C19 is predicted to be mostly stabilized by its interaction with the αT5 loop.

## Discussion

In this study, 55 colchicine derivatives were screened for their binding properties to tubulin isotype βIII. The research work was aimed at identifying alternative compounds able to overcome colchicine's well-known limitations. After the docking of all compounds to the target isotype of tubulin, a molecular dynamics simulation of 1 ns was performed on each generated receptor-ligand complex. The obtained trajectories were analyzed considering the deviations of the compounds from the colchicine's starting pose, using the RMSD, and the binding energy evaluated with the MM-GBSA method. All compounds were characterized by low RMSD values, except for compound C19, which showed high deviations (RMSD = 0.47 nm). This evidence suggests a different particular pose for this derivative. From the affinity analysis we found out that the binding energies for compounds C19, C20, C29, and C48 are similar to that for colchicine and higher than those found for most other ligands. These results indicate that C19 is a promising compound to be further investigated and experimentally validated. Its specific binding to tubulin is characterized by a different conformational organization and dynamics in the tubulin binding site with high affinity. RMSD analysis indicates that C19 is able to be accommodated in the binding site by moving toward more favorable poses for interaction with the αT5 loop. This feature is less pronounced by colchicine. Moreover, the buried surface between C19 and the tubulin isotype βIII, which measures the available area for the binding, is greater than the one exhibited with colchicine, confirming a higher stability of C19 in the binding site. Finally, the ligand binding to the αT5 loop may affect its secondary structure toward a more structured arrangement. Therefore, a compound able to influence the αT5 loop structure could affect the dynamics of the entire microtubule.

The above mentioned evidences might be of a significant interest given that the αT5 loop is a key player region in the colchicine binding site and for intra-dimer contacts (Ravelli et al., 2004). Nonetheless, previous literature (Bueno et al., 2018) already highlighted the importance of the αT5 loop, identified as relevant for the binding of a promising anti-proliferative compound (Bueno et al., 2018).

In conclusion, our study clarifies some features characterizing the βIII tubulin binding mode of a promising novel 4-chlorine thiocolchicine derivative, which differs profoundly from that known for colchicine. The specific interaction of compound C19 with the αT5 loop is a promising feature that could be related to an increased destabilizing activity of the ligand with respect to microtubule dynamics. Moreover, this unique behavior exhibited in complex with the βIII tubulin isotype is of primary importance since this isotype is overexpressed in cancer cells, while very insignificantly represented in most normal cells and also implicated in drug resistance (Katsetos et al., 2003; Kamath et al., 2005; Seve, 2005; Sève and Dumontet, 2008; Leandro-García et al., 2010). In light of these results, C19 or similar compounds, as promising candidates able to possibly overcome some colchicine's drawbacks, deserve further investigations, including biological toxicity assessment and cancer cell cytotoxicity experiments to prove its specificity and selectivity for βIII isotype of tubulin.

## Data Availability Statement

The raw data supporting the conclusions of this article will be made available by the authors, without undue reservation, to any qualified researcher.

## Author Contributions

MD, JT, and AH conceived the research. LP, AR, and GG did the molecular dynamics simulations. LP, AR, GG, and GK analyzed and rationalized the data. All authors wrote the paper and critically commented to the manuscript, read, and approved the final manuscript.

### Conflict of Interest

The authors declare that the research was conducted in the absence of any commercial or financial relationships that could be construed as a potential conflict of interest.
