# Supplementary material for: In silico Investigations of the Mode of Action of Novel Colchicine Derivatives Targeting β-Tubulin Isotypes: A Search for a Selective and Specific β-III Tubulin Ligand
Source: Front Chem. 2020 Feb 21;8:108. doi: 10.3389/fchem.2020.00108 (PMC7047339; doi:10.3389/fchem.2020.00108)
Supplement: Supplementary file 1 [file Data_Sheet_1.PDF]

Supporting Information

S1. Human βIII Tubulin Model Validation and Conformational Dynamics

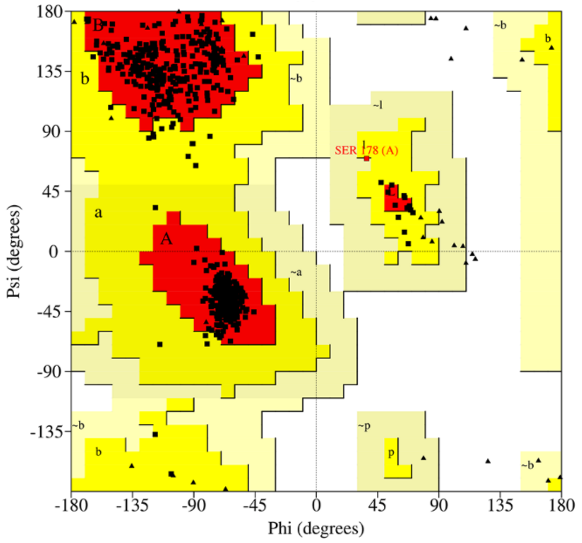

Figure S1. Ramachandran plot of βIII tubulin model generated by homology modeling.

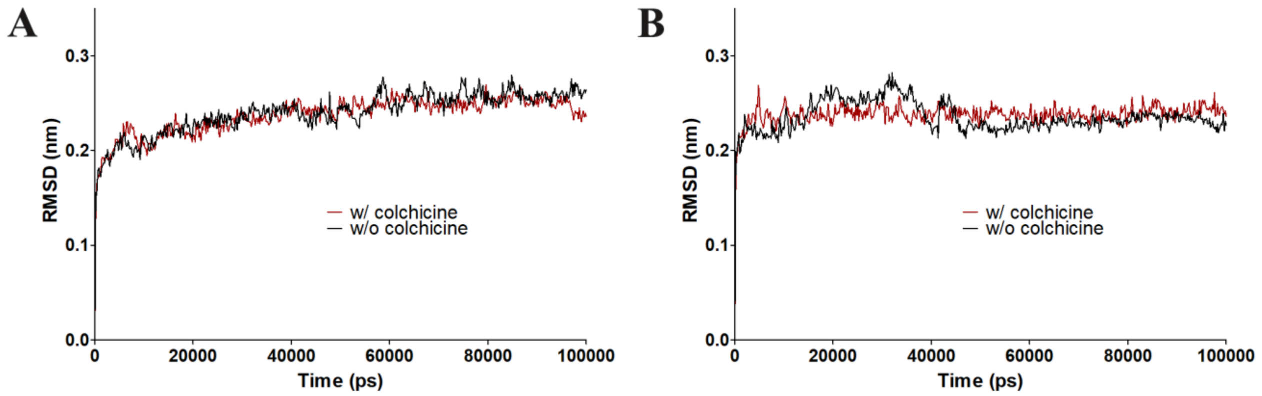

Figure S2. RMSD of α (A) and β (B) monomers during the 100 ns simulations in presence (red) and in absence (black) of colchicine.

S2. Ensemble Docking and Binding Energy Calculation

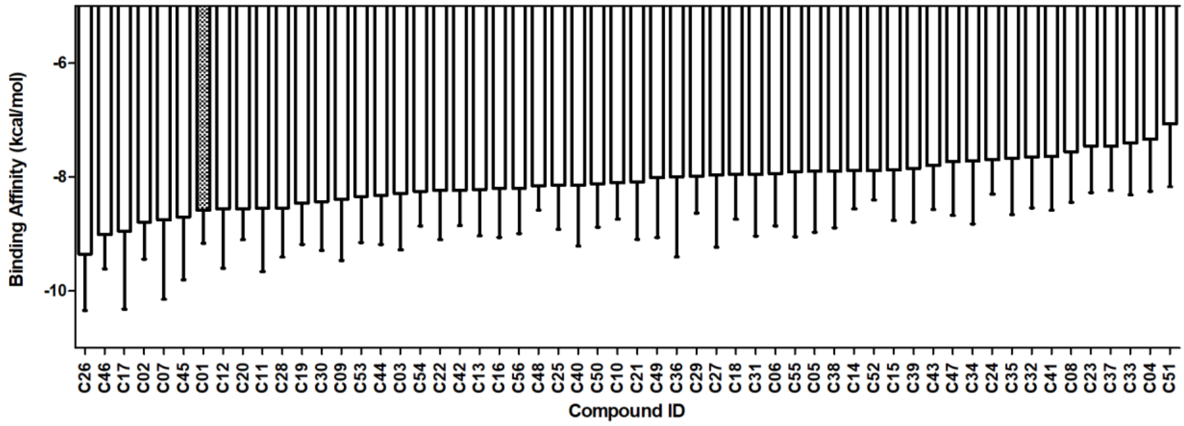

Figure S3. Binding affinities between all compounds and isotype βIII calculated by VINA; colchicine (C01) is highlighted using a different pattern.

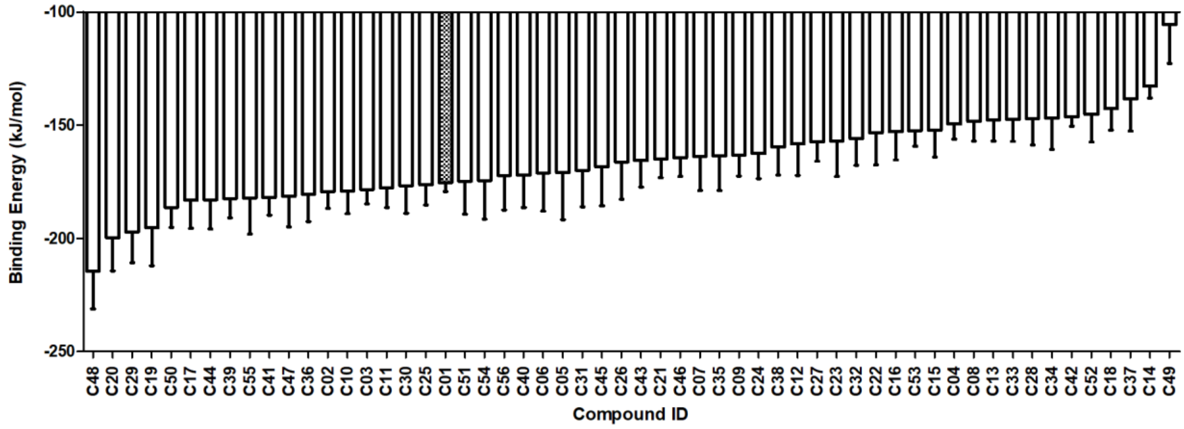

Figure S4. Refined Binding energies between all compounds and isotype βIII evaluated by MM-GBSA method after a short MD run (1ns); colchicine (C01) is highlighted using a different pattern

S3. Colchicine and C19 bound to βIII Human Tubulin

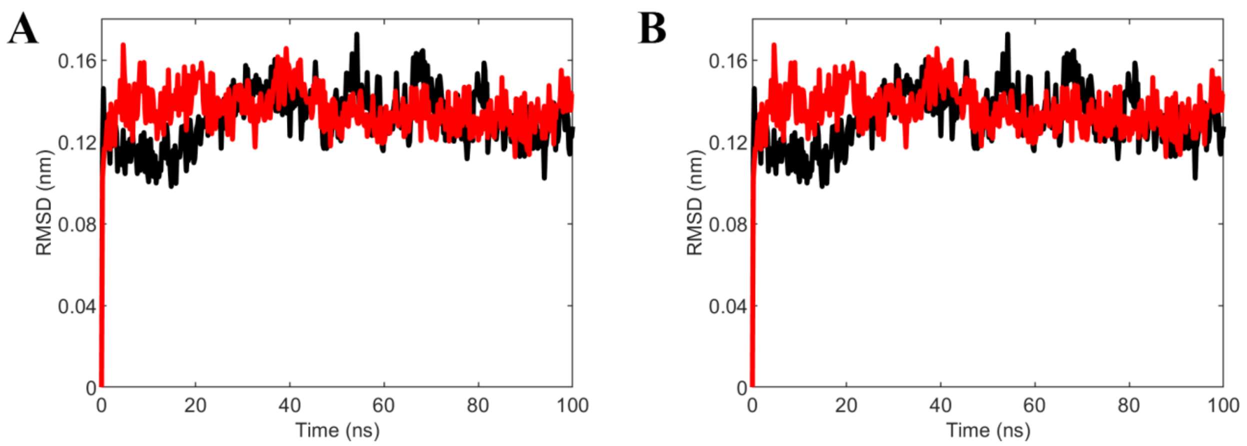

Figure S5. RMSD of the colchicine binding site (residues within 1 nm from the ligand) from its starting position for colchicine (A) and compound C19 (B). In black the first replica and in red the second one.

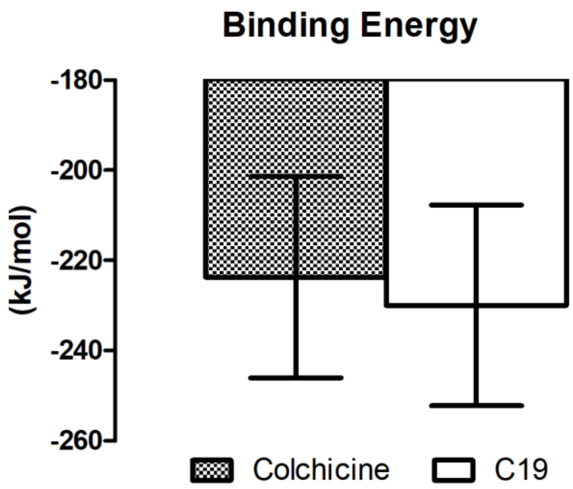

Figure S6. Binding energies of colchicine and compound C19 for isotype βIII evaluated with the MM-GBSA method during the last 50 ns of MD simulations.

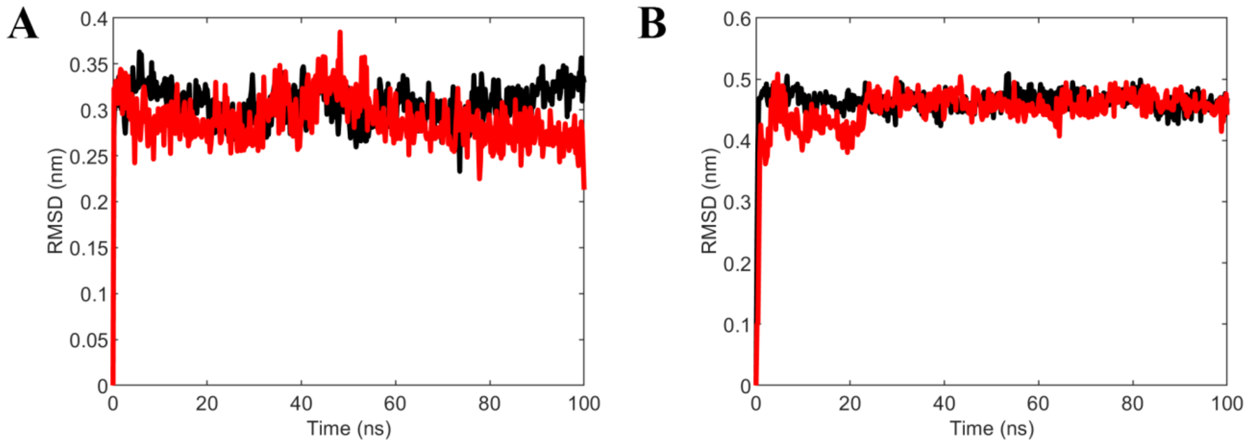

Figure S7. RMSD of the ligands from their starting position for colchicine (A) and compound C19 (B). In black the first replica and in red the second one.

Supplementary Movie 1 and Supplementary Movie 2. First and second replica trajectories of colchicine binding site (residues within 1 nm from the ligand). Colchicine is represented in green, compound C19 in red and their relative αT5 loops are consistently colored. Movies show that compound C19 is able to further move apart to reach a more favorable position for the interaction with the above-mentioned loop, increasing the buried surface.
